# Experimental Analysis of Dynamic Effects of FRP Reinforced Masonry Vaults

**DOI:** 10.3390/ma8125445

**Published:** 2015-11-27

**Authors:** Marco Corradi, Antonio Borri, Giulio Castori, Kathryn Coventry

**Affiliations:** 1 Department of Mechanical and Construction Engineering, Northumbria University, Wynne-Jones Building, Newcastle upon Tyne NE1 8ST, UK; kathryn.coventry@northumbria.ac.uk; 2 Department of Engineering, University of Perugia, 92 Via Duranti, Perugia 06125, Italy; antonio.borri@unipg.it (A.B.); giulio.castori@unipg.it (G.C.)

**Keywords:** composite materials, vibration, brickwork masonry, mechanical testing

## Abstract

An increasing interest in the preservation of historic structures has produced a need for new methods for reinforcing curved masonry structures, such as arches and vaults. These structures are generally very ancient, have geometries and materials which are poorly defined and have been exposed to long-term historical movements and actions. Consequently, they are often in need of repair or reinforcement. This article presents the results of an experimental study carried out in the laboratory and during on-site testing to investigate the behaviour of brick masonry vaults under dynamic loading strengthened with FRPs (Fiber Reinforced Polymers). For the laboratory tests, the brick vaults were built with solid sanded clay bricks and weak mortar and were tested under dynamic loading. The experimental tests were designed to facilitate analysis of the dynamic behaviour of undamaged, damaged and reinforced vaulted structures. On-site tests were carried out on an earthquake-damaged thin brick vault of an 18th century aristocratic residence in the city of L’Aquila, Italy. The provision of FRP reinforcement is shown to re-establish elastic behavior previously compromised by time induced damage in the vaults.

## 1. Introduction

Masonry arches and vaults, common to historic buildings, have been predominantly designed to resist vertical static loads, hence dynamic horizontal loads induced by earthquakes often cause significant damage or the collapse of these structural elements.

Considerable progress has been made in understanding arch and vault behavior in the last three decades [1,2,3,4] and several repair and/or strengthening methods have been proposed for re-establishing and enhancing the static and dynamic performance of curved masonry structures. However, these proposals are promoted on the basis of static testing alone, potentially due to a reluctance to address the complexities of dynamic analysis. However, it is apparent that failure to consider the efficacy of repair work to historic vaulted structures under dynamic behavior will constrain a rigorous evaluation of the most appropriate design solutions and promote the employment of ineffective implementation strategies to fortify these structures. All potential loading conditions acting on historic vaulted structures must be considered if our global building heritage is to be preserved.

This investigation of historic vaulted structures, proposes addressing the complexity of applied loading conditions through the adoption of composite materials. FRP-materials present a solution for the retrofitting and seismic fortification of historical buildings located in earthquake zones. The application of these materials to situations where an increase in strength is to be balanced against minimising an increase in mass, has become widespread for more than two decades; the static efficiency of these repairs being demonstrated in the laboratory and through numeric modelling techniques [5,6,7].

Fiber reinforced polymers (FRP) reinforcements provide engineers the option to explore interesting repair techniques. This includes the possibility of easy reinforcement removal and replacement, while maximizing the benefits resulting from the low self-weight and high strength of these fibres, facilitating conservation through maintaining the boundary conditions of the original structure and not increasing the original mass.

The FRP reinforcement of masonry vaults is incapable of preventing masonry from cracking or repairing an existing crack, but it does transmit the tension force between the two sides of the crack, stitching the crack together. Hence, hinges may form, but cannot open, since the tension force is transmitted by the reinforcement in lieu of the cracked masonry, *i.e.*, the tension force bypasses the crack and transfers directly to the reinforcement. This means that reinforcing the vault’s extrados or intrados, effectively prevents collapse mechanisms from occurring, forcing such structures to fail by other failure modes (*i.e.*, crushing, sliding, debonding or FRP rupture).

Increases in loading capacity ranging from 30% to 600% have been recorded from static tests on reinforced and unreinforced structures. This has been noted in many experimental and analytical studies in the last two decades on masonry arches [8,9,10,11], masonry barrel vaults [12,13,14], cross vaults and domes [15,16,17]. While these researchers predominantly focus on the static behaviour of masonry structures strengthened with composite materials, studies based on dynamic behaviour would more accurately simulate the load application conditions that induce failure, while facilitating the analysis of the dynamic parameters of a vaulted structure achieved after strengthening interventions employing FRPs. Experimental studies of the dynamic behavior of historic masonry structures reinforced with composite materials are rare [18,19,20] and the investigation of FRP reinforcement applied to vaulted structures under dynamic test conditions has not yet been addressed, this study attempts to address these omissions in the research conducted to date.

## 2. Experimental Program and Vault Details

Tests were carried out within the laboratory and on site, with the aim of analyzing the dynamic behavior of undamaged, damaged and FRP-reinforced vaulted masonry structures.

Three full scale masonry vaults were constructed in laboratory for the purpose of this investigation from solid clay bricks and weak cement-based mortar. Two of the vaults (BV1 and BV2) were of a barrel vault design and were constructed from identical brickwork specification. Vault RV3 was a rib barrel thin vault. Insertion of ribs in barrel vaults allows for thickness reduction without a significant decrease in the structure’s stiffness. Two ribs with a cross section of 25 cm × 12 cm were constructed for the barrel thin vault.

**Figure 1:**
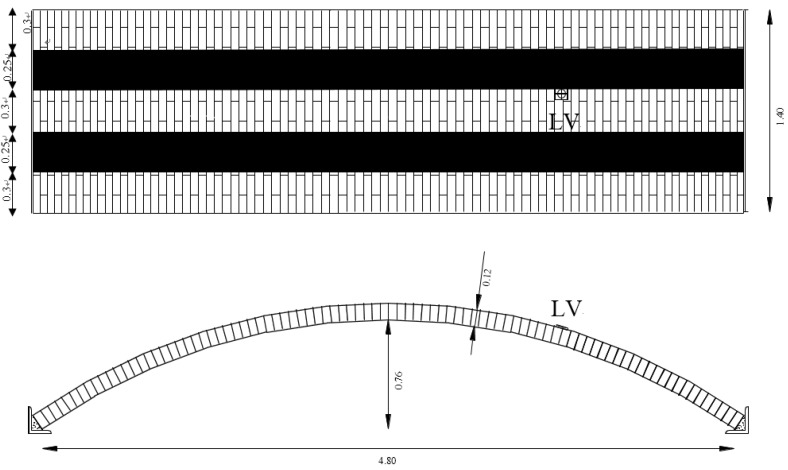
Configuration of the two barrel vaults (LV point is the position of the target point of the laser beam of the vibrometer) (dimensions in m).

The experimental vaults were constructed with a span of 4.80 m, a rise of 0.76 m and a depth of 1.40 m, respectively. The wall thickness for vaults BV1 and BV2 was 12 cm (Figure 1), while a thickness of 5.5 cm was adopted for RV3 (thin vault).

**Figure 2:**
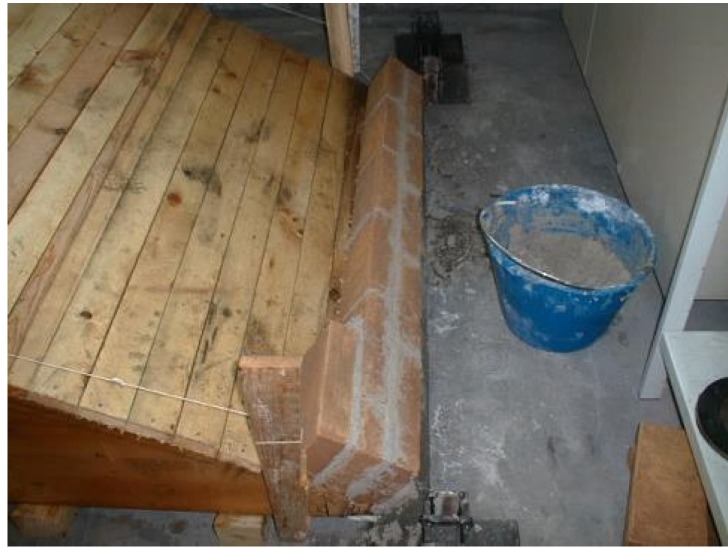
Construction detail for the masonry barrel vaults springing (of BV1-type).

The specimens were built on a wooden framework, with the barrel vaults springing constructed from L shaped steel profile and effectively fixed to the floor. A triangular concrete cast block was utilised along the vault’s depth to commence the masonry work with full bricks for both springings (Figure 2).

Each of the three vaults were simply supported at the base, while dynamic behaviour was evaluated under the following structural vault configurations: (a) undamaged vault; (b) damaged vault; (c) repaired vault with FRP-materials. The damage of the vaults arose from the formation of three hinges and subsequent cracks in the mortar joints.

The tests carried out on the damaged and repaired vaults utilised strengthening material in the form of uni-directional glass fabrics (GFRP). Two 250 mm wide fibre strips bonded at the vaults extrados at a distance of 550 mm apart, were employed throughout the experimental program in the laboratory. The surfaces of the repaired vaults were manually cleaned in order to remove any residual dust prior to the application of the composite strips. The surface was then pre-impregnated with an epoxy primer to improve bonding at the interface. A layer of epoxy putty was applied to fill the voids between the bricks; another layer of the bi-component epoxy resin was applied at the surface, before and after, the placement of the fabric strip. The placement of the strips ensured that the fibres lay perpendicular direction to the cracks and became embedded in the resin through the application of a light pressure. The curing time for the FRP reinforcement was seven days at room temperature, in accordance with the resin product specification.

**Figure 3:**
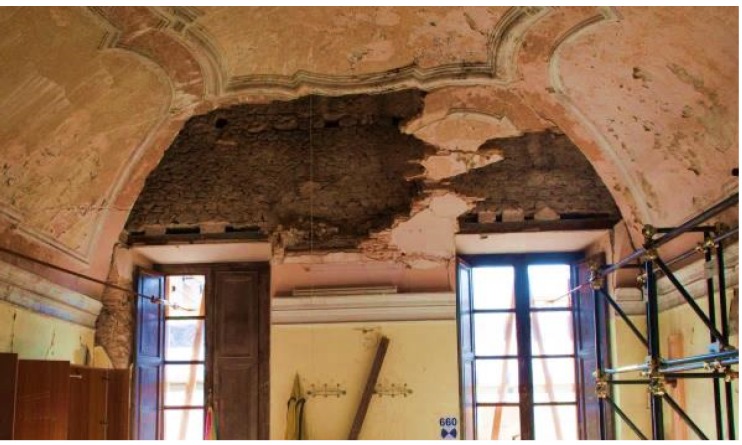
The thin vault tested in the city of L’Aquila.

Field tests were carried out on the vaults of an historical building in L’Aquila, Italy. This building, an aristocratic residence dating back to 18th century with important decorative features (frescos, marble window seals, fire-places, decorative mouldings, *etc.*), was heavily damaged by the 2009 earthquake. The large rooms of the main floor were characterized by thin tile vaulted ceilings, partially filled at the extrados with light in-fill material to ensure structural stability (Figure 3). This is a typical arrangement for brick vaults in the city of L’Aquila: very large in-folio (thin) brick vaults partially filled with building rubble. These structural masonry elements usually exhibit weak behavior under seismic loads, highlighting the necessity for reinforcement and retrofitting.

**Figure 4:**
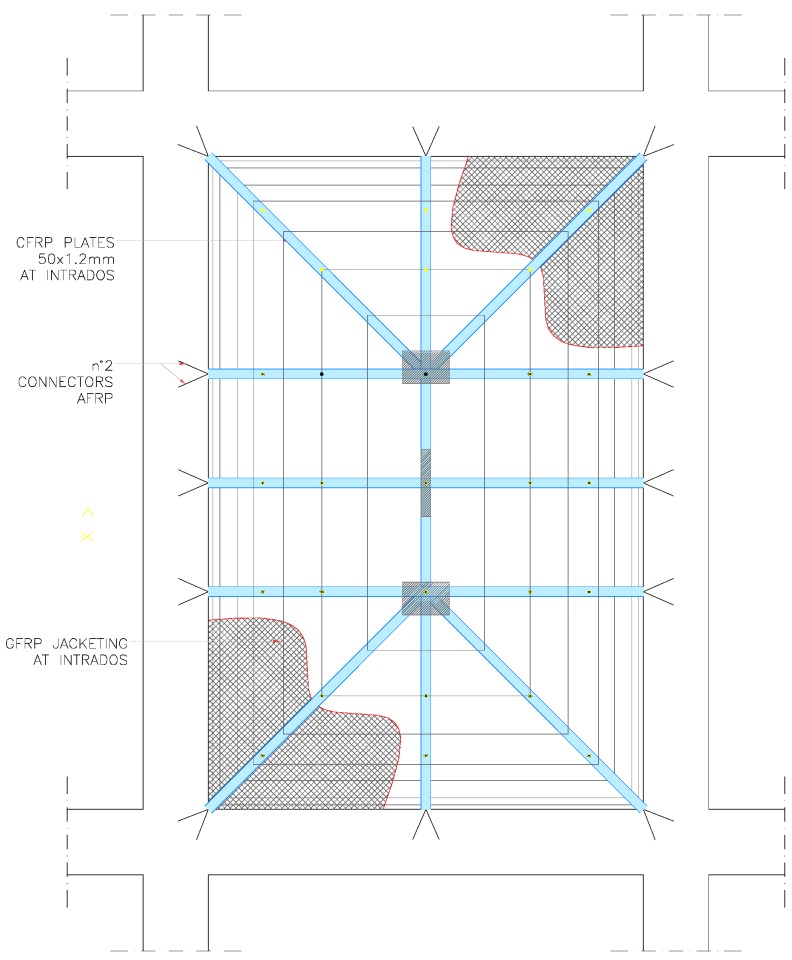
Layout of reinforcement for the L’Aquila vault.

The tests in this study were carried out on a large damaged thin vault. Dynamic tests were performed before and after the application of the composite materials. Reinforcement was applied at the vault’s intrados due to the presence of a high-historic value ancient floor over the vault, thereby preventing any kind of work at the vault’s extrados. The vault was not decorated with frescos, which facilitated the application of the reinforcement. However, the imposition of constraints by the Italian body for preservation and conservation of historic monuments (Soprintendenza per i Beni Architettonici e Paesaggistici) did not allow an extensive use of polymeric adhesives (epoxy resins) to bond the composites at the vault intrados, despite being aimed at guaranteeing the non-destructive removability of reinforcement and compatibility with historic masonry. For this reason, a GFRP textile reinforced mortar coupled with a non-polymeric cement-based matrix was used and applied over the entire vault intrados surface (Figure 4).

**Figure 5:**
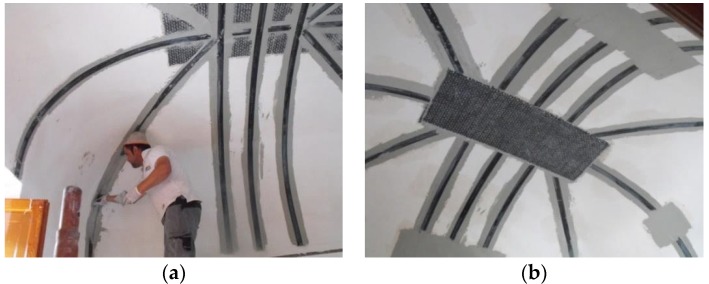
Reinforcement application: (**a**) CFRP strips; (**b**) CFRP sheet at vault crown.

In order to improve the structural behavior of the vault, Carbon FRP strips (CFRP) were applied at the vault’s intrados over the GFRP mesh, to form an internal FRP-reinforced arch (Figure 5a). The intention was to create a structural element with a similar structural mechanism to that of an umbrella: the thin-tile GFRP reinforced vault represented the fabric of the umbrella while the CFRP strips acts as the spokes. In addition, local reinforcement in form of CFRP sheet crossing was applied over the GFRP mesh. The CFRP sheets were bi-directional with a dimension of 500 mm × 600 mm and a density of 300 g/m^2^ (Figure 5b).

It should be noted that Aramidic fibres (AFRP) connectors in the form of spikes were driven into the bricks at the abutments, resulting in a brick penetration of approximately 80 mm. This was done for only the CFRP strips resulting in a penetration into the bricks of approximately 80 mm, ensuring adequate connection between the CFRP strips and the vault.

### 2.1. Material Specification

For laboratory tests, solid clay bricks of nominal dimension 5.5 cm × 12 cm × 25 cm were adopted in the construction of the vaults. Thirty bricks, tested according to American Society for Testing Material (ASTM) C67 [21], gave a mean compressive strength of 20.99 MPa and root-mean-square deviation of 1.87 MPa.

The mortar mix design used was established as 6:2.5:0.5 (sand:lime:cement). Eighteen compression tests have been conducted on the mortar in accordance with ASTM C349 [22]. The mortar had a compression strength of 1.92 MPa and a Coefficient of Variation (CoV) of 18.4%. Nine mortar prisms were tested in bending according to ASTM C348 [23]; the prisms’ dimensions were 40 mm × 40 mm × 160 mm. Bending strength was 0.29 MPa and CoV (27.5%).

In order to measure the mechanical properties of the brickwork masonry, two wallettes with dimensions 509 mm × 335 mm × 115 mm were built for each vaulted specimen for evaluating masonry compression strength and Young’s modulus. The weight density of this masonry was 1657 kg/m^3^, with average compressive strength and average elastic modulus values of 6.17 MPa (CoV = 20.7%) and 3634 MPa (CoV = 17.1%), respectively. The fibre adopted in this investigation consisted of a uni-directional woven fabric made of Alkaline Resistant (AR) Glass fiber (GFRP). The fabric was impregnated by an epoxy resin.

For laboratory and in-situ tests mechanical properties of CFRP strips, GFRP textile and CFRP sheet and AFRP spikes, as reported in the producer’s data sheet, are shown in Table 1. CFRP strips were 50 mm × 1.2 mm in cross section and made by pultrusion.

**Table 1:** Mechanical characteristics of fiber reinforced polymer (FRP)-materials.

Property	AR GFRP	CFRP Strips	GFRP Textile	AFRP Spikes
Fiber orientation	Unidir.	Unidir.	Bidir.	Unidir.
Young’s Modulus (GPa)	65 **	205	74 **	100 **
Weight density (kg/m^2^)	0.6	1.71	0.22	–
Tensile strength (MPa)	1700 **	3252	2875 **	2905 **
Thickness (mm)	0.23 *	1.2	0.048 *	–
Elongation at failure (%)	2.8	1.6	2.9	1.44

* dry fabric, ** using dry fabric thickness.

## 3. Test Results

The method of data analysis employed in this study is referred to as the peak-amplitude or peak-picking method. This method is appropriate for the examined masonry structures with well-separated modes [1,24].

### 3.1. Laboratory Tests

**Figure 6:**
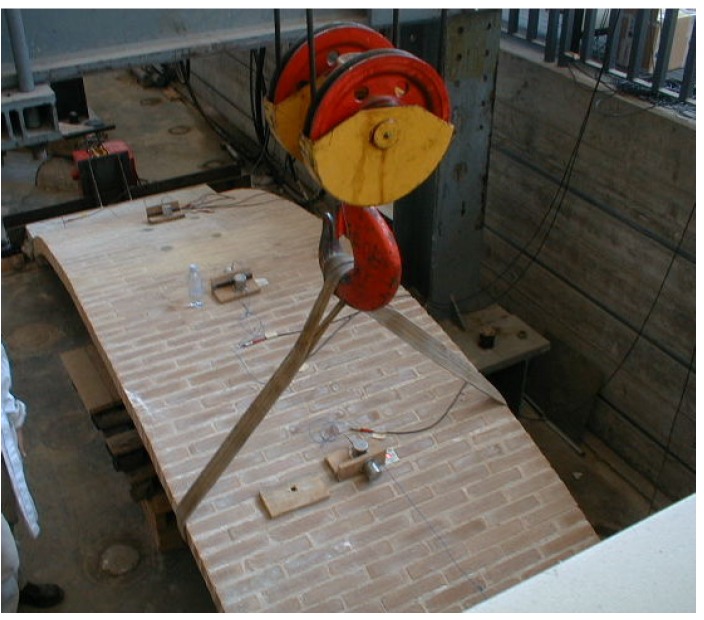
Raising of the vault to allow free vibrations.

For the laboratory tests, the dynamic excitation is achieved through the use of a sling which allows the “vertical raising” of the vault followed by the release of the sling to enable the vault to vibrate (Figure 6) until rest. Individual resonance peaks are detected on the Frequency Response Function (FRF) plot and the frequency of maximum response is taken as the mode natural frequency.

In the case of specimen BV1, the sling was applied at left haunch and the excitation method allowed the measurement of the natural modal frequencies, particularly the first mode frequency. Measurement of vibrations of the vaults have been recorded using a Polytec Doppler laser-vibrometer model OFV 3001-OFV 303 (Polytec GmbH, Waldbronn, Germany) with CPU OFV-600 at the center point of the “raised” haunch along a direction with a slope of 40° on the horizontal plane. Results are partially reported in [25]. From the experimental investigations on specimens BV2 and BV3, with the excitation applied at one haunch, the results are presented in Table 2.

**Table 2:** Natural frequencies under free oscillation.

Specimen	Undamaged		Damaged		Strengthened	
	1st Mode (Hz)	2nd Mode (Hz)	1st Mode (Hz)	2nd Mode (Hz)	1st Mode (Hz)	2nd Mode (Hz)
BV1	11.25	–	10.50	–	12.50	–
BV2	10.00	–	6.25	10.62	9.37	18.75
BV3	6.25	13.00	3.50	9.00	8.50	15.50

**Figure 7:**
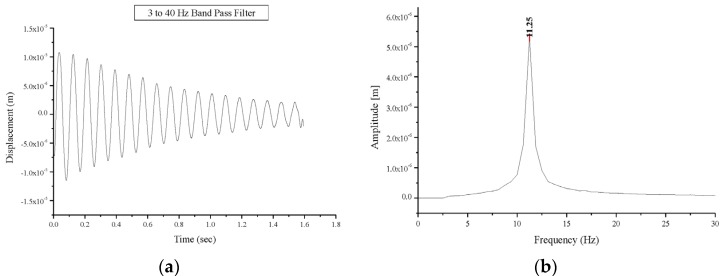
Free oscillations response of the undamaged vault BV1: (**a**) displacement versus time; (**b**) Fourier transform: amplitude versus frequency.

Testing of the masonry vaults occurred 45 days post-construction. The first modal frequencies values of 11.25 Hz and 10.00 Hz were recorded for BV1 and BV2 respectively (Figure 7) while a much lower natural frequency value, 6.25 Hz, up to 40% lower, was recorded for BV3. These frequency values can be also compared with those obtained from a numerical simulation: frequencies of 10.66 and 9.62 Hz were found from the numerical simulation for BV1- and BV3-type vaults, respectively. For a BV1-type vault, these values are in a very good agreement with experimental ones. The difference in dynamic behavior illustrated by the first-mode natural frequency of BV3 when compared with BV1 and BV2, can be explained by the difference in vault thickness and the presence of the ribs.

A simple 3D linear finite element model has been used to deepen the dynamic behaviour of the vault. The geometry is the same of the experimentally tested specimens and the vault body is created by an Ansys [26] software using Solid 65 elements (three-dimensional eight-node hexahedron isoparametric elements). For the boundary conditions in the finite element model of the vault, all translations are fixed at the vault imposts, but rotations are allowed.

For the sake of simplicity, both elastic and perfectly plastic behavior were adopted: (a) with infinite ductility and (b) with isotropic behavior hypotheses for the material. It must be underlined that such assumptions do not take into account two important aspects of masonry at failure, the first related to the damaging behavior of joints, the latter related to the well-known higher masonry horizontal in-plane strength with respect to the vertical one, essentially due to brick staggering. Nevertheless, these aspects cannot be taken into account easily. On the other hand, the assumption of a zero tensile strength with frictional behavior is widely accepted [27] for the analysis of masonry constructions. Within this approach, a Mohr–Coulomb type failure criterion with tension cut-off type behavior was assumed for masonry. This failure criterion, initially adopted for concrete, accounts for both cracking and crushing failure modes through a smeared model. In particular, the masonry brittle behavior was here defined by means of only two constants: *f*_t_ (uniaxial tensile strength) and *f*_c_ (uniaxial compressive strength).

**Figure 8:**
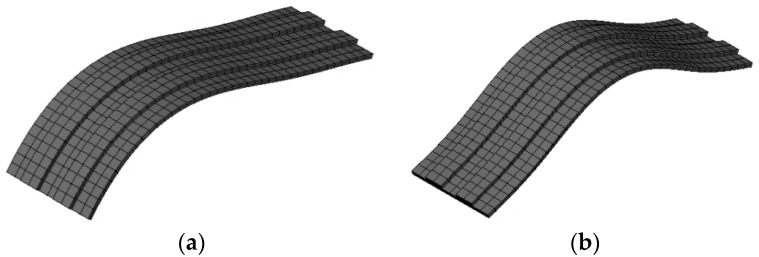
Numeric simulation: the first (**a**) and second (**b**) mode of vibration (BV3).

The main aim of this simple model was only to determine the natural frequencies and modes of vibrations. Figure 8 shows the first two natural modes of vibration of BV3. For undamaged vaults, this analysis demonstrated that the experimental value of first natural frequency is very similar to the numerical one: the first natural frequency from the model was 11.12 Hz (compared to 11.25 and 10 Hz measured for BV1 and BV2, respectively). For damaged vaults, the results of numerical and experimental tests highly differed for the difficulty of modelling the vault damage and the hinges. This difference (in terms of first natural frequency value) reduced for strengthened vaults (numerical 11.84 Hz, average experimental 10.93 Hz) due to the mitigation of non-linear effects.

Damping values were also calculated for all the three tested masonry vaults (Table 3). The value of the damping α, has taken into consideration the time range (*t_i+_*_1_, *t_i_*) of approximate 0.4 s and also the tests with the sling placed at the left haunch. For an underdamped system:

(1) $$x\left(t\right)=-Xe^{-\alpha t}$$

The value of the damping α is given by:

(2) $$\alpha =\frac{1}{\left(t_{i+1}-t_{i}\right)}\ln\left(\frac{x_{i}}{x_{i+1}}\right)$$

*x_i_* and *x_i+_*_1_ are the peaks of the displacements *x(t)* respectively within the time range *t_i+_*_1_, *t_i_*.

**Table 3:** Recorded damping values from free oscillations.

Specimen	Undamaged (s^−1^)	Damaged (s^−1^)	Strengthened (s^−1^)
BV1	1.28	1.50	1.47
BV2	1.25	2.08	1.75
BV3	1.22	1.87	1.15

**Figure 9:**
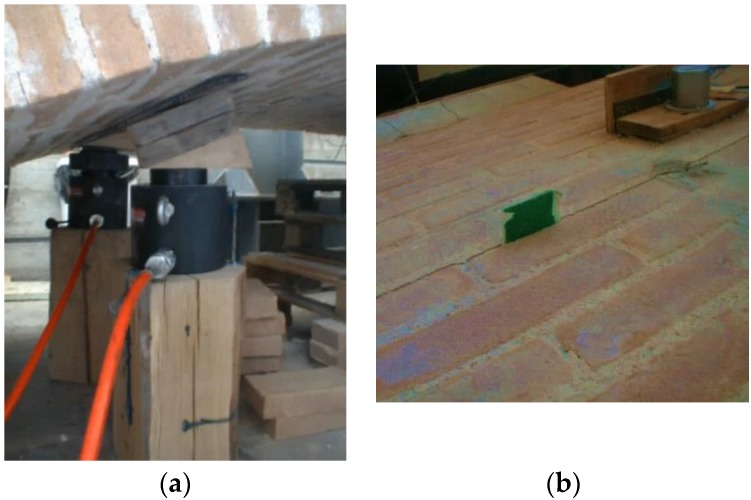
(**a**) Application of hydraulic cylinders to promote damage; (**b**) Hinge formation.

Free oscillation tests were also performed on the damaged vaults. Damage was caused by a vertical load through the application of two hydraulic cylinders and a spreader beam under one haunch (Figure 9). The static vertical loading was increased until hinges formed at three locations along the curved length of the vault; one hinge occurred around the vault springing, another hinge formed over the position of the vertical load, and the third hinge formed in the opposite haunch 11 brick lengths from the crown (for BV1-type vault).

**Figure 10:**
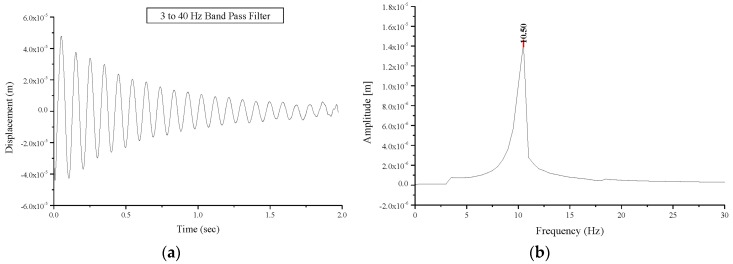
Free oscillations’ response of the damaged vault BV1: (**a**) displacement versus time; (**b**) Fourier transform: amplitude versus frequency.

The global behavior of the damaged vaults was predominantly elastic allowing for analysis of the vault’s behavior via free oscillation tests, as previously done for the undamaged vault. A decrease in the frequencies of the first natural mode was recorded in all tests carried out on the damaged vaults compared to the values recorded for undamaged vaults. Natural frequencies values of first and second mode for the tested masonry vaults are presented in Table 2. The first natural mode frequency value for specimen BV1 experiences a decrease from 11.25 Hz (undamaged vault) to 10.50 Hz (damaged vault) as shown in Figure 10, while a decrease from 10.00 Hz to 6.25 Hz and from 6.25 Hz to 3.50 Hz was recorded for specimens BV2 and BV3, respectively. The decrease in the first-mode natural frequency is a consequence of the damage sustained. This value depends on the positions of the hinges and the extent of structural damage. This explains the different values (10.50 and 6.25 Hz) recorded for vaults BV1 and BV2. It must be highlighted that if damage does not progress, this value remains constant with time. Similarly, an increase in the damping values was experienced in the damaged vaults compared to the undamaged vaults. The damping values are summarized in Table 3. Corresponding values were recorded for undamaged vaults (average value 1.25 s^−1^). The damage resulted in an increase in the damping value between 17.1% and 66.4%.

**Figure 11:**
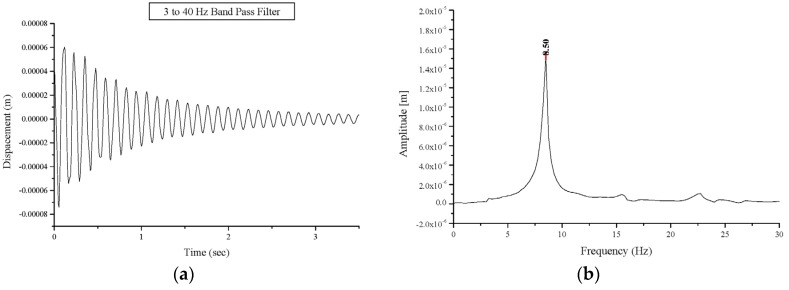
Free oscillations’ response of the strengthened vault BV3: (**a**) displacement versus time; (**b**) Fourier transform: amplitude versus frequency.

**Figure 12:**
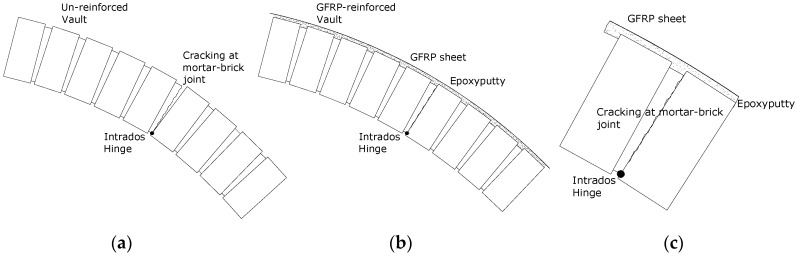
(**a**) Hinge opening at intrados for the un-reinforced vault; (**b**) GFRP reinforced vault; (**c**) detail at the hinge after the application of the reinforcement.

The test procedure, applied to the undamaged and damaged vaults was repeated on strengthened vaults, and the results are presented in Table 2 and Table 3. Due to the strengthening, an increase in the frequencies of the first natural mode compared to those for damaged vaults was recorded. The first mode frequency values for the strengthened vaults were almost the same as that of the undamaged vaults. The first natural mode frequency for specimen BV3 after strengthening, reached the value of 8.50 Hz compared the 6.25 Hz and 3.50 Hz for the same vault in the undamaged and the damaged configurations, respectively (Figure 11). The cracks where the three hinges formed are still present, but tensile stresses are now transferred to the GFRP reinforcements (Figure 12).

Strengthening with GFRP sheets has resulted in a decrease of the damping value (Table 3) for the repaired vault compared to the vault that is damaged but has had no strengthening interventions. However, the application of the GFRP at the vault extrados has not re-established the original damping values due to the presence of the mortar joint cracks. This could be considered as an additional advantage to be gained from strengthening. Reinforcement can greatly enhance the tensile strength of masonry while re-establishing the original dynamic response of the undamaged structure.

Consideration of the behavior of the rib masonry barrel vault illustrates the natural frequency of the strengthened specimen to be higher than in the control undamaged vault. This may be explained by the increased masonry stiffness between the ribs, resulting from the application of fibre strips. A high decrease in damping was suggested in the damping value following the strengthening application.

### 3.2. On-site Tests

A further dynamic investigation has been carried out on-site on a thin brick vault to validate the efficiency of the reinforcing technique. Dynamic tests were performed on the vault before and after the application of reinforcement at the intrados. Free vault oscillations were induced by a blow from a sledgehammer. The tests were conducted over a period of four days, as a preliminary investigation into the dynamic characteristics of the vault. A full modal survey was not conceived, the study only served to identify the prominent modes of vibration. It was expected that a mildly non-linear response of the masonry vault would be observed due to the presence of cracks at the sides of the un-strengthened damaged vault.

A 2 kg sledgehammer instrumented with a piezoelectric force transducer was used for the forced excitation of the vault. The response of the structure was measured using three low-frequency Monitran MTN/7100 accelerometers with nominal sensitivity of 5.39 mV/g.

**Figure 13:**
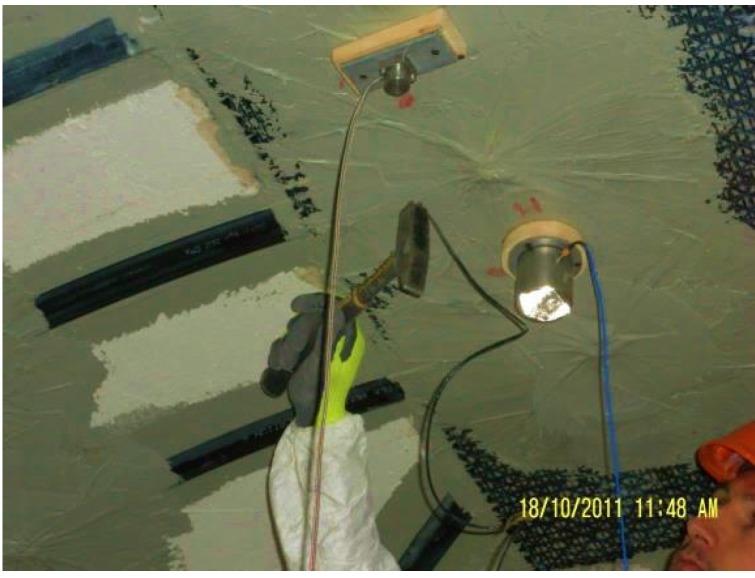
Sledge hammer applied to the crown of the vault intrados.

Both force and response signals were filtered using a 16th order low-pass Butterworth filter with a cut-off frequency of 200 Hz. Accelerometers mounted on steel blocks were attached to the masonry vault with an adhesive. Hammer excitation was concentrated on the vault key-stone, as it was almost level, and, therefore, easy to balance on (Figure 13). The magnitude of the accelerations were recorded at the vault haunches on a direction perpendicular to the vault surface at that point. The Frequency Response Function (FRF) for each location was obtained from an average of ten hammer blows for a spectral resolution of 0.0625 Hz.

**Figure 14:**
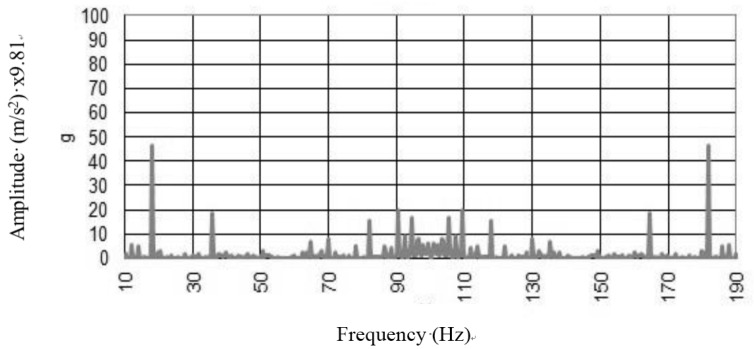
Free oscillations’ response to the *in situ* test on the damaged vault.

Typical FRF spectra for un-reinforced vault (Figure 14) are presented showing their component magnitudes. The damage to the vault promoted an inelastic dynamic response and any natural frequency could be recorded for the vault. Dynamic pounding between closely spaced parts of the vault resulted in the recording of high peaks for the amplitude of acceleration, but any dynamic analysis could be executed.

**Figure 15:**
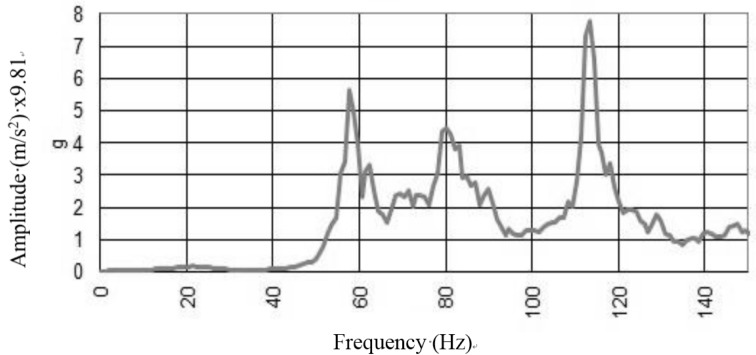
Free oscillations response to the *in situ* test on the reinforced vault.

For the reinforced vault, an elastic behavior was restored and the vault exhibited a clear dynamic behavior (Figure 15). Modal peaks in the spectra indicated the presence of a number of modes of vibration below 120 Hz. The well-defined peaks represent individual modes, and their repeatability across the full spectral set facilitated a modal analysis. Re-establishing the elastic behavior of the vault through application of reinforcement is considered to be a positive result of the investigation.

## 4. Conclusions

The effectiveness of FRP-reinforcement of vaulted and arch masonry structures has been demonstrated through static tests by several research investigations evidenced by the rich bibliography presented in this paper. In advancing this work, this research considers dynamic behavior before and after the application of FRP reinforcement.

The strengthening of masonry vaults through FRPs have highlighted some limitations as well as advantages. The dynamic tests have been carried out in laboratory on three full-scale masonry barrel vaults constructed from two different masonry solid-brick brickworks (two barrel vaults and one rib barrel vault) and on-site on a damaged thin-brick vault before and after reinforcement.

The results of the laboratory tests detail the dynamic behaviour of the masonry vaults in the un-damaged, damaged and strengthened configurations. In particular, it is noted that the FRP strengthening operations promote the approximate re-establishment of the value of frequency of first natural mode, prior to damage. The strengthening is partially able to restore the dynamic behaviour of the undamaged vaults, without changing the constraint conditions and increasing the dead loading to the structure. The benefits of this non-invasive reinforcement technique are identified as having particular application to retrofitting, comparing favorably to traditional invasive techniques which can result from the practice of constructing a reinforced concrete layer on top of the original extrados. FRPs can be removed mechanically upon application of heat.

Detachment of the composites from the masonry surface, was never observed during the test procedures employed. Even for vault BV3, the mono-directional GFRP sheet remained attached to the masonry surface. However, further research on the long-term adhesion and of the mechanical properties of composite materials for particular site conditions, is necessary.
